# A redox-mediator pathway for enhanced multi-colour electrochemiluminescence in aqueous solution†

**DOI:** 10.1039/d1sc05609c

**Published:** 2021-12-15

**Authors:** Emily Kerr, David J. Hayne, Lachlan C. Soulsby, Joseph C. Bawden, Steven J. Blom, Egan H. Doeven, Luke C. Henderson, Conor F. Hogan, Paul S. Francis

**Affiliations:** Institute for Frontier Materials, Deakin University Geelong Victoria 3220 Australia emily.kerr@deakin.edu.au; School of Life and Environmental Sciences, Faculty of Science, Engineering and Built Environment, Deakin University Geelong Victoria 3220 Australia paul.francis@deakin.edu.au; Department of Chemistry and Physics, La Trobe Institute for Molecular Science, La Trobe University Melbourne Victoria 3086 Australia

## Abstract

The classic and most widely used co-reactant electrochemiluminescence (ECL) reaction of tris(2,2′-bipyridine)ruthenium(ii) ([Ru(bpy)_3_]^2+^) and tri-*n*-propylamine is enhanced by an order of magnitude by *fac*-[Ir(sppy)_3_]^3−^ (where sppy = 5′-sulfo-2-phenylpyridinato-*C*^2^,*N*), through a novel ‘redox mediator’ pathway. Moreover, the concomitant green emission of [Ir(sppy)_3_]^3−^* enables internal standardisation of the co-reactant ECL of [Ru(bpy)_3_]^2+^. This can be applied using a digital camera as the photodetector by exploiting the ratio of R and B values of the RGB colour data, providing superior sensitivity and precision for the development of low-cost, portable ECL-based analytical devices.

## Abstract 2

A water-soluble Ir(iii) complex is shown to enhance the ‘remote’ mechanism of the most widely used co-reactant ECL reaction of tris(2,2′-bipyridine)ruthenium(ii) with tripropylamine.

## Graphical abstract

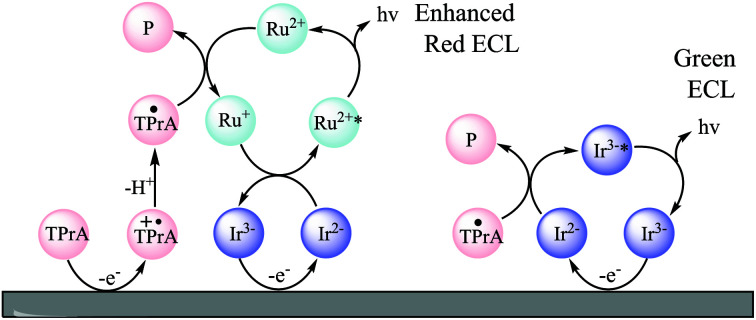

## Introduction

Electrochemiluminescence (ECL) is a highly sensitive mode of detection^1,2^ frequently employed in clinical diagnostic assays such as those of the Roche *cobas e* automated immunoassay system.^3^ Conventional ECL bioassays incorporate an electrochemiluminophore (label) and a ‘co-reactant’ that enables the light-producing reaction to be initiated at a single applied potential in aqueous solution.^4^ The majority of ECL applications, and all commercial ECL technologies, use the tris(2,2′-bipyridine)ruthenium(ii) ([Ru(bpy)_3_]^2+^) luminophore (or a closely related derivative) and tri-*n*-propylamine (TPrA) co-reactant.^5^ Efforts to enhance the sensitivity of this fundamental ECL detection system have included the development of ‘poly-Ru^2+^’ labels, exploiting dendrimers^6^ or nanoparticles,^7^ but the diminishing increase in ECL intensity with the number of metal centres, and greater background signals due to higher non-specific binding has limited their use in clinical diagnostics.^3^ Alternatively, numerous Ir(iii) complexes have shown great promise as high quantum yield luminophores for ECL in organic media,^8^ and their diverse electrochemical potentials and emission wavelengths have enabled the preliminary development of potential-resolved and multi-colour ECL systems,^2,9^ towards the long-held goals of multiplexed^9–11^ and internally standardised, ratiometric^12^ ECL detection.‡‡Other reported ratiometric ECL approaches (predominantly applied with nanoparticle-based luminophores) exploit phenomena such as competition for co-reactants^13^ or resonance energy transfer between luminophores,^14^ or involve internal standardisation with distinct ECL systems (*e.g.*, [Ru(bpy)_3_]^2+^ or luminol).^15^ Internal standardisation was also demonstrated for the co-reactant ECL of a [Ru(bpy)_3_]^2+^ derivative and 2-(dibutylamino)ethanol (DBAE) using the internal electrochemical reference signal of methylene blue.^16^^13–16^ The poor solubility and/or reduced ECL performance of many Ir(iii) complexes in aqueous solution, however, has restricted their analytical application.

To enhance the water solubility, ECL intensity, and/or binding specificity of Ru(ii) and Ir(iii) complex electrochemiluminophores, researchers have incorporated 4,7-diphenyl-1,10-phenanthroline-disulfonate ligands^17,18^ or synthesised new ligands with polar functional groups such as methanesulfonate, tetraethylene-glycol and saccharides.^3,19,20^ Comparisons with [Ru(bpy)_3_]^2+^ in the ‘ProCell’ buffer solution (containing TPrA co-reactant and a surfactant) used in commercial ECL immunoassay system, however, have revealed that only a few outperform the conventional luminophore.^18,20,21^ One of the most promising candidates, an Ir(iii) complex containing two sulfonate-bearing phenylphenanthridine derivatives and a phenylisoquinoline-based ligand for bioconjugation, exhibits 3–4 fold greater ECL intensity than the Ru(ii) complex.^3^ Multi-colour ECL systems incorporating Ir(iii) complexes have to date been predominantly limited to organic solvents^11,22–24^ and polymer-ionic-liquid gels,^25^ but have been coupled with assays in aqueous solution through closed bipolar electrochemistry,^24,26^ or by loading the luminophores into polystyrene beads that support the assay, followed by their release in the organic solvent for detection.^27^

The most commonly used Ir(iii) complex in multi-colour ECL is *fac*-tris(2-phenylpyridinato)iridium(iii) (*fac*-Ir(ppy)_3_),^10,11,26–33^ due to the large differences in its electrochemical potentials and emission wavelengths compared to [Ru(bpy)_3_]^2+^, and the effective quenching of its ECL at high overpotentials under certain conditions when TPrA is used as the co-reactant,^28,29^ which enables the complete resolution of its emission from other luminophores through the applied potential. Moreover, the addition of Ir(ppy)_3_ to the annihilation ECL reaction of [Ru(bpy)_3_]^2+^ in acetonitrile was shown to enhance (∼25-fold) the emission from the Ru(ii) complex, through the ‘mixed annihilation ECL’ reaction of the reduced [Ru(bpy)_3_]^+^ with oxidised [Ir(ppy)_3_]^+^ (reaction (1)).^31^

1 [Ru(bpy)_3_]^+^ + [Ir(ppy)_3_]^+^ → [Ru(bpy)_3_]^2+^* + Ir(ppy)_3_

**Fig. 1:**
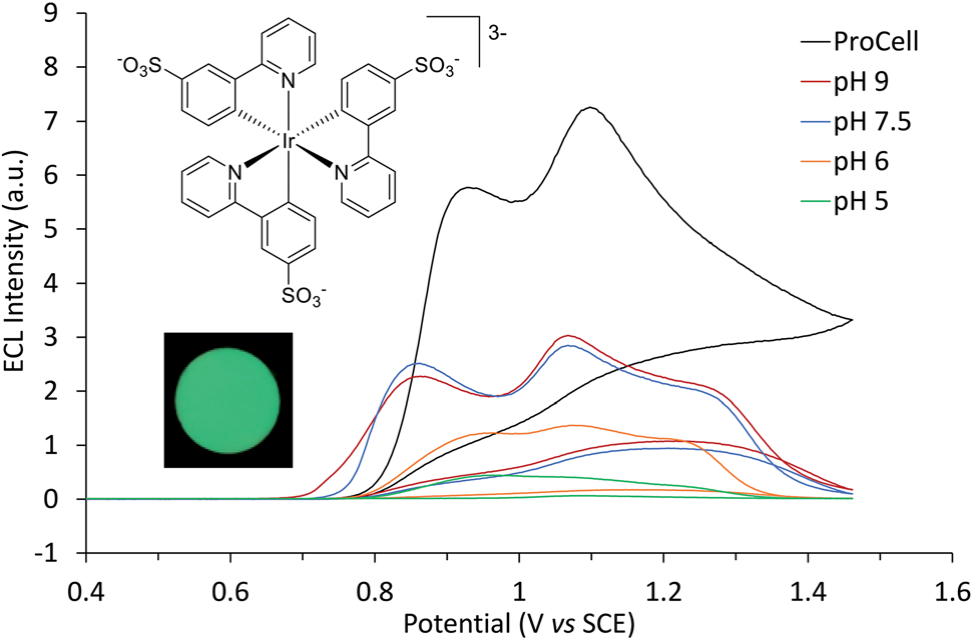
ECL intensity of [Ir(sppy)_3_]^3−^ (0.25 mM) in 0.1 M phosphate buffer at specified pH with 100 mM TPrA, or in ProCell solution, over a voltametric scan from 0 V to 1.46 V *vs.* SCE (and back to 0 V) at 0.1 V s^−1^. The inset photograph shows the green ECL at the working electrode surface (0.2 mM [Ir(sppy)_3_]^3−^ in 0.1 M phosphate buffer (pH 7.5) with 100 mM TPrA; applied potential 0.89 V *vs.* SCE). Camera settings: ISO 3200, *f*/2.8, shutter time 16 s.

Considering that the multiple reaction pathways of the co-reactant ECL of [Ru(bpy)_3_]^2+^ and TPrA involve oxidation and/or reduction of the Ru(ii) complex,^5^ we sought to use Ir(ppy)_3_ as an alternative means to enhance this most widely applied system. The Ir(ppy)_3_ complex is insoluble in aqueous solution, but Wenger *et al.*^34,35^ recently outlined a convenient synthetic strategy to add a sulfonate group to each ligand of the intact complex, which they used for photoredox catalysis in aqueous solution. Herein, we adopt this synthetic approach to explore [Ir(sppy)_3_]^3−^ (Fig. 1) as a novel luminophore for aqueous ECL, which enables the first multi-colour ECL from a mixture of metal complexes in aqueous solution. We then demonstrate that the [Ir(sppy)_3_]^3−^ complex can enhance the ECL of [Ru(bpy)_3_]^2+^ with TPrA co-reactant by over an order of magnitude, while providing an unprecedented means for internal standardisation of this important ECL system. Finally, we show that the enhancement and internal standardisation can be exploited when using a digital camera as the photodetector (*via* the ratio of R and B values of RGB colour data), providing a new approach to overcome the reproducibility and sensitivity limitations of low-cost, portable analytical devices with ECL detection.

## Results and discussion

### Electrochemistry and photoluminescence

The photophysical and electrochemical properties of [Ir(sppy)_3_]^3−^ are compared to those of Ir(ppy)_3_, [Ru(bpy)_3_]^2+^, and a previously reported^20^ water-soluble Ir(iii) complex electrochemiluminophore, [Ir(ppy)_2_(pt-TEG)]^+^, in Table 1. [Ir(sppy)_3_]^3−^ readily dissolved at 5 mM in phosphate buffer solution (PBS) and exhibited a reversible oxidation (Fig. S1†) at 0.79 V *vs.* SCE, marginally higher than the oxidation potential of Ir(ppy)_3_ in acetonitrile (0.71 V *vs.* SCE^36^). The sulfonated derivative was insoluble in acetonitrile (at 0.1 mM) and we therefore determined the potential of the [Ir(sppy)_3_]^3−/4−^ couple (*E*^0^′ = −2.48 V *vs.* SCE) in dimethylformamide. The [Ir(sppy)_3_]^3−^ complex exhibited green photoluminescence in aqueous solution (*λ*_max_ = 515 nm; Fig. S2†) at slightly higher energy than Ir(ppy)_3_ in acetonitrile (*λ*_max_ = 520 nm (ref. 36)). A small difference in emission energy was also observed in the low temperature (85 K) emission spectra in 4 : 1 ethanol : methanol (Table 1). Wenger and co-workers reported an excited state oxidation potential for [Ir(sppy)_3_]^3−^ at −1.89 V *vs.* SCE,^37^ based on the ground-state oxidation potential (*E*^0^′(M^+^/M)) and the energy of the emissive triplet state (*E*_0–0_) estimated from the short-wavelength edge of the emission (at 10% of the maximum intensity) at room temperature. Our measurement of both ground state potentials and the *E*_0–0_ estimated from the *λ*_max_ of the low-temperature emission spectrum enabled calculation of the excited state oxidation and reduction potentials as −1.82 V and 0.57 V *vs.* SCE.

**Table 1:** Photophysical and electrochemical data

	[Ru(bpy)_3_]^2+^	[Ir(ppy)_2_(pt-TEG)]^+^	[Ir(sppy)_3_]^3−^	Ir(ppy)_3_
Abs. (*λ*_max_)/nm^a^	243, 285, 452	251, 376	245, 271, 360	242, 280, 380 (ref. 38)
PL (*λ*_max_)/nm^a^^,^^b^	622	476, 505	515	520
PL (*λ*_max_; 85 K)/nm^b^^,^^c^	581, 629 (ref. 20)	471, 506, 536 (ref. 20)	481, 516	494, 532 (ref. 36)
*E*_0–0_/eV^d^	2.13	2.63	2.58	2.51
QY (*Φ*_PL_)/%	6.3^e^^,^^41^	14^f^^,^^20^	72.9^e^^,^^34^	70^g^^,^^42^, 90^h^^,^^43^, 97^i^^,^^44^, 99^g^^,^^38^
*E* ^0^′(M^+^/M)/V *vs.* SCE	1.06 (Aq)^j^, 1.27 (ACN)^l^^,^^20^	1.08 (Aq)^j^, 1.24 (ACN)^l^^,^^20^	0.79 (Aq)^j^, 0.77^k^^,^^37^, 0.76 (DMF)^m^	0.71 (ACN)^l^^,^^36^
*E* ^0^′(M/M^−^)/V *vs.* SCE	−1.35 (ACN)^l^^,^^20^	−1.82 (ACN)^l^^,^^20^	−2.01 (DMF)^m^	−2.29 (ACN)^l^^,^^36^
*E* ^0^(M^+^/M*)/V *vs.* SCE^n^	−0.86	−1.39	−1.82 (−1.89)^37^	−1.78
*E* ^0^(M*/M^−^)/V *vs.* SCE^o^	0.78	0.81	0.57	0.24
ECL *λ*_max_/nm	623	504	516	520
ECL *I*_rel_ (10 mM TPrA)^p^^,^^q^	100^s^	29.2 ± 1.8^t^	0.27 ± 0.01^t^	0.40^g^^,^^38^
ECL *I*_rel_ (100 mM TPrA)^p^^,^^r^	100^s^	24.6 ± 2.2^t^	0.18 ± 0.01^t^	—
ECL *I*_rel_ (ProCell)^p^^,^^r^	100^s^	36.5 ± 1.5^t^	1.09 ± 0.03^t^	—

a Metal complexes at 10 μM in water (or acetonitrile for Ir(ppy)_3_) at ambient temperature.

b Corrected for the change in instrument sensitivity over the wavelength range.

c Metal complexes at 5 μM in ethanol : methanol (4 : 1) at 85 K.

d Energy gap between the zeroth vibrational levels of the ground and excited states, estimated from the *λ*_max_ of the low-temperature emission spectrum.

e Aqueous solution; deaerated.

f Aqueous (ProCell) solution; aerated.

g Acetonitrile; deaerated.

h Dichloromethane, deaerated.

i 2-Methyltetrahydrofuran (2-MeTHF), deaerated.

j Converted to SCE from Ag/AgCl (3.5 M KCl) by subtracting 40 mV.^45^

k Converted to SCE from Ag/AgCl (sat. KCl) by subtracting 46 mV.^45^

l Converted to SCE from Fc^+^/Fc^0^ (in ACN with 0.1 M TBAPF_6_ electrolyte) by adding 0.38 V.^46^

m Converted to SCE from Fc^+^/Fc^0^ (in DMF with 0.1 M TBAPF_6_ electrolyte) by adding 0.47 V.^46^

n Estimated using *E*^0^′(M^+^/M) − *E*_0–0_(M–M*).

o Estimated using *E*^0^′(M/M^−^) + *E*_0–0_.

p Integrated ECL intensity upon application of 1.2 V (*vs.* Ag/AgCl) for 10 s, measured using CCD spectrometer.

q Metal complex at 100 μM.

r Metal complex at 10 μM.

s By definition.

t Error represents standard deviation of five replicates.

### Electrochemiluminescence

[Ir(sppy)_3_]^3−^ exhibited green ECL (*λ*_max_ = 516 nm, Fig. S3†) with TPrA as a co-reactant, which increased in intensity from pH 5 to 7.5 in 0.1 M PBS (Fig. 1). The ECL intensity with 10 mM TPrA was 0.27% that of [Ru(bpy)_3_]^2+^ with the same co-reactant, which is similar to the relative intensity of Ir(ppy)_3_ (0.40%) to [Ru(bpy)_3_]^2+^ with 10 mM TPrA in acetonitrile.^38^ Greater ECL from [Ir(sppy)_3_]^3−^ (1.09% that of [Ru(bpy)_3_]^2+^) was obtained in ‘ProCell’ solution, a commercially available ECL reaction matrix composed of 180 mM TPrA, surfactant (0.1%) and preservative in 0.3 M PBS at pH 6.8, developed for commercial ECL analysers.^21^

**Fig. 2:**
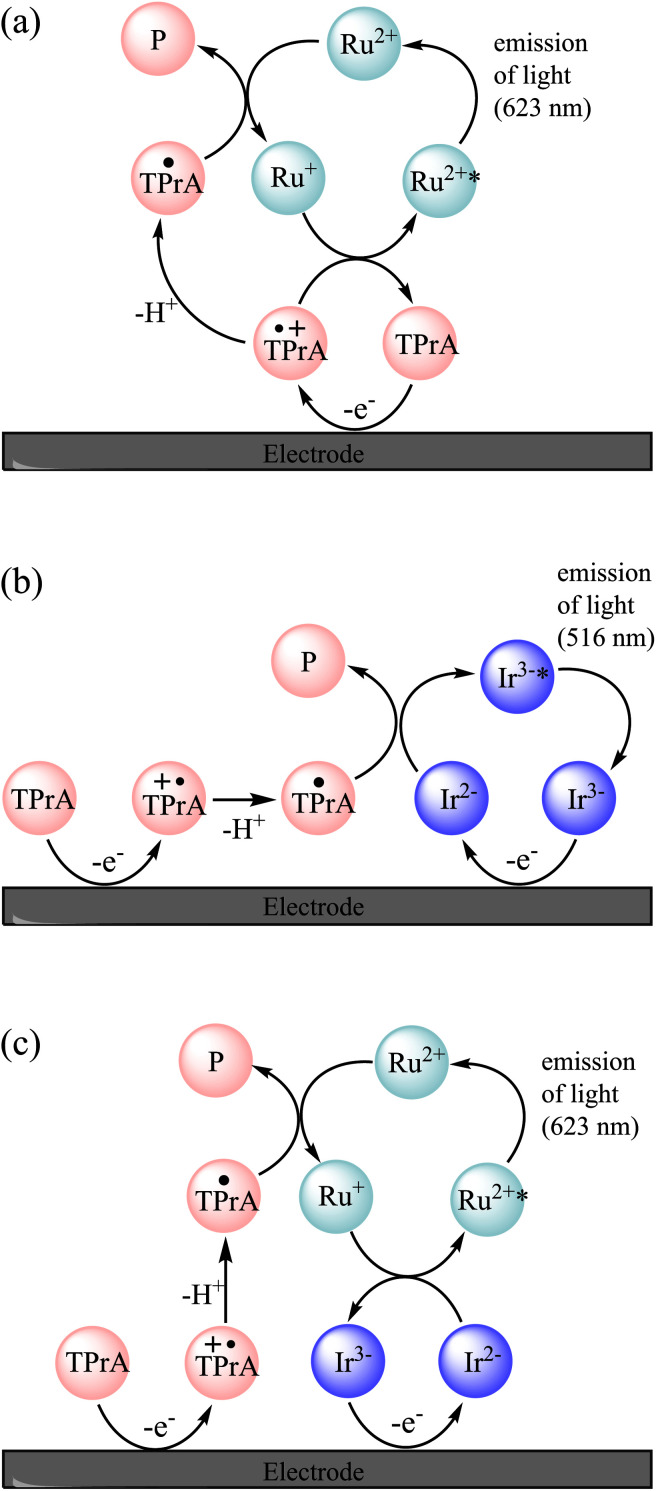
Mechanisms involved in the co-reactant ECL of a mixture of [Ru(bpy)_3_]^2+^ (Ru^2+^) and [Ir(sppy)_3_]^3−^ (Ir^3−^) with TPrA co-reactant, in circumstances in which TPrA and [Ir(sppy)_3_]^3−^ (but not [Ru(bpy)_3_]^2+^) are oxidised. This occurs at potentials between 0.81 V and 1.06 V *vs.* SCE, and at higher potentials if the [Ru(bpy)_3_]^2+^ luminophore is not close enough to the electrode for its direct oxidation (*e.g.*, when immobilised in bead-based assays). (a) The unenhanced ‘remote’ co-reactant ECL of [Ru(bpy)_3_]^2+^. An analogous pathway is not feasible for [Ir(sppy)_3_]^3−^ because TPrA˙ cannot reduce that complex. (b) The ‘direct’ co-reactant ECL of [Ir(sppy)_3_]^3−^. As the [Ru(bpy)_3_]^2+^ is not oxidised under these conditions, it cannot generate light *via* this pathway. (c) The enhanced ECL of [Ru(bpy)_3_]^2+^. The reaction of [Ir(sppy)_3_]^2−^ and [Ru(bpy)_3_]^+^ can generate [Ru(bpy)_3_]^2+^* and [Ir(sppy)_3_]^3−^ (but not [Ru(bpy)_3_]^2+^ and [Ir(sppy)_3_]^3−^*).

Possible pathways to attain the [Ir(sppy)_3_]^3−^* excited state can be drawn from those of the well-known co-reactant ECL^5^ of [Ru(bpy)_3_]^2+^ and TPrA (reactions (2)–(10), where TPrA˙^+^ is Pr_3_N˙^+^ and TPrA˙ is Pr_2_NC˙HCH_2_CH_3_) with consideration of the corresponding redox potentials and excited state energy of [Ir(sppy)_3_]^3−^ (Table 1).^39^ The TPrA˙ radical (*E*^0^_ox_ ≈ −1.7 V *vs.* SCE) can reduce [Ru(bpy)_3_]^2+^ (*E*^0^′ = −1.35 V *vs.* SCE) but not [Ir(sppy)_3_]^3−^ (*E*^0^′ = −2.01 V *vs.* SCE), which means that reactions (7)–(9) are not feasible for the [Ir(sppy)_3_]^3−^ complex. Moreover, unlike [Ru(bpy)_3_]^3+^, [Ir(sppy)_3_]^2−^ cannot oxidise TPrA (*E*^0^′ ≈ 0.79–0.91 V *vs.* SCE^5^), which removes the ‘catalytic route’^5^ shown as reaction (4). The light-producing pathway for [Ir(sppy)_3_]^3−^ with TPrA as a co-reactant is therefore limited to reactions (2), (3), (5), (6) and (10) (Fig. 2b).^40^

2 TPrA → TPrA˙^+^ + e^−^

3 M → M^+^ + e^−^

4 M^+^ + TPrA → M + TPrA˙^+^

5 TPrA˙^+^ → TPrA˙ + H^+^

6 M^+^ + TPrA˙ → M* + P

7 M + TPrA˙ → M^−^ + P

8 M^+^ + M^−^ → M* + M

9 M^−^ + TPrA˙^+^ → M* + TPrA

10 M* → M + *hv*

The two ECL intensity maxima observed when increasing the applied potential (Fig. 1) are reminiscent of the ‘two waves’ of ECL of the [Ru(bpy)_3_]^2+^ or certain Ir(iii) complexes such as [Ir(bt)_2_(pt-TEG)]^+^, with the TPrA co-reactant.^5,20,47^ This arises from the two distinct pathways to the excited state^5,20^ involving electrooxidation of (i) TPrA only (reactions (2), (5), (7), (9) and (10); as depicted in Fig. 2a), which is referred to as the ‘remote’ mechanism,§§Zanut *et al.*^40^ recently revealed an additional pathway involving the oxidative C–N bond cleavage of TPrA to form dipropylamine radicals, but for the purposes of this discussion, we group those short-lived intermediates with TPrA˙^+^ and TPrA˙.^40,48^ or (ii) both TPrA and the metal complex (reactions (2)–(6), (8) and (10)), referred to as the ‘direct’ mechanism.^48^ However, as the first pathway is not feasible for [Ir(sppy)_3_]^3−^, this effect is not involved here. In acetonitrile, the co-reactant ECL of Ir(ppy)_3_ with TPrA has been found to ‘switch-off’ at high overpotentials, ascribed to oxidative quenching by the TPrA˙^+^ intermediate (reaction (11)).^28,29^ The ECL of an analogue containing an electron withdrawing fluorine group (Ir(F-ppy)(ppy)_2_) was considerably less quenched.^30^ Partial quenching could also be anticipated in the co-reactant ECL of [Ir(sppy)_3_]^3−^ (as its excited state is a less powerful reductant than Ir(ppy)_3_*; Table 1), which may explain the local intensity minima at ∼1 V in Fig. 1.

11 M* + TPrA˙^+^ → M^+^ + TPrA

### Multi-colour and ratiometric ECL

**Fig. 3:**
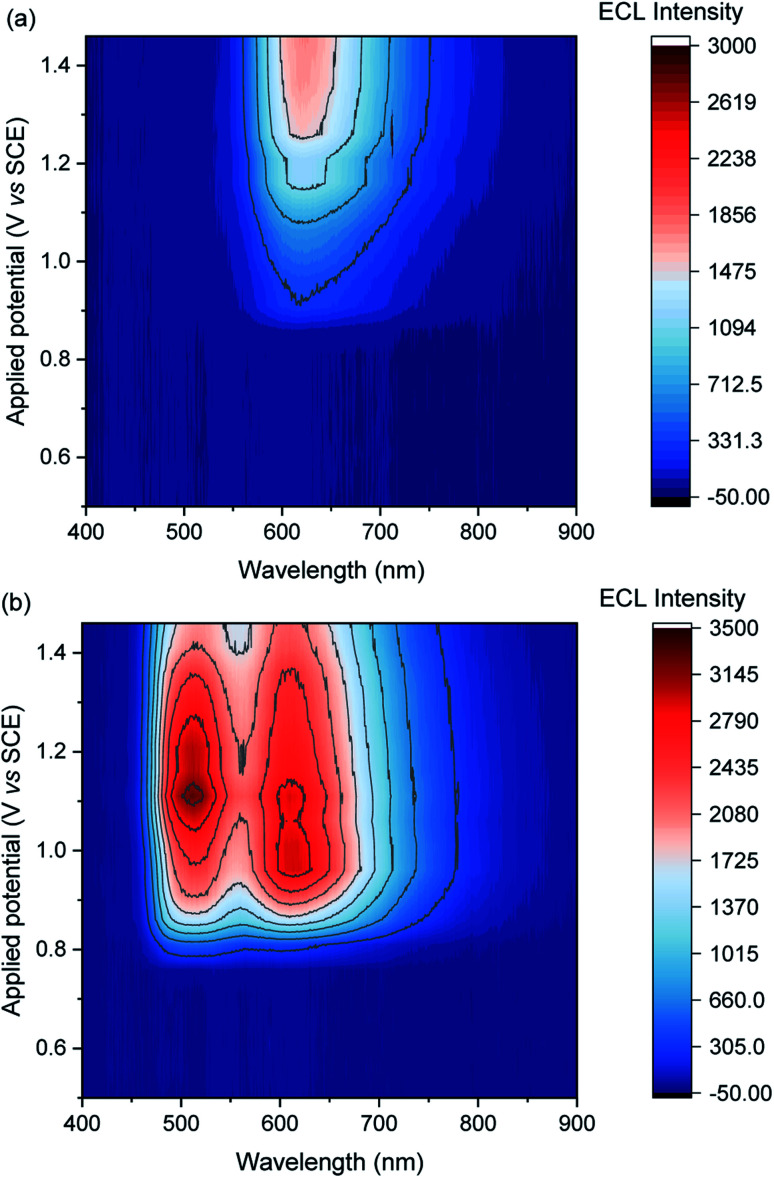
Contour plots of ECL *vs.* wavelength and applied potential for (a) [Ru(bpy)_3_]^2+^ (0.75 μM) or (b) a mixture of [Ru(bpy)_3_]^2+^ (0.75 μM) and [Ir(sppy)_3_]^3−^ (150 μM), in ProCell solution, prepared by applying the potentials (10 s chronoamperometric pulses) in 50 mV intervals. The contour plot for [Ir(sppy)_3_]^3−^ (150 μM) is shown in Fig. S5.† A plot of the ECL intensity at 620 nm *vs.* applied potential is shown in Fig. S7.†

We previously demonstrated switching between the green and red ECL of Ir(ppy)_3_ and a [Ru(bpy)_3_]^2+^ derivative within the same acetonitrile solution (with TPrA co-reactant) simply by changing the applied potential.^28^ This potential-resolved ECL^2^ exploited the distinct *E*^0^′(M^+^/M) of the metal complexes and the quenching of Ir(ppy)_3_* at high overpotentials. The [Ir(sppy)_3_]^3−^ complex exhibits similar photophysical and electrochemical characteristics to Ir(ppy)_3_ (Table 1), but as noted above, its co-reactant ECL with TPrA is not strongly quenched at high overpotentials in aqueous phosphate buffer or ProCell solution (Fig. 1). For a mixture of [Ru(bpy)_3_]^2+^ and [Ir(sppy)_3_]^3−^ in ProCell solution, the ECL of both luminophores ‘switches on’ at approximately 0.85 V (Fig. 3 and S4†), through the mechanisms shown in Fig. 2a and b, and was observed over the remainder of the potential range (to 1.46 V *vs.* SCE).

To obtain similar ECL intensities from the two luminophores in the mixture (Fig. 3 and S5†), a much greater concentration of [Ir(sppy)_3_]^3−^ than [Ru(bpy)_3_]^2+^ was required, due to their relative co-reactant ECL efficiencies, and a considerable enhancement of the [Ru(bpy)_3_]^2+^ ECL when combined with [Ir(sppy)_3_]^3−^. Energy transfer from [Ir(sppy)_3_]^3−^* to [Ru(bpy)_3_]^2+^ was ruled out as a major source of this enhancement because: (i) there is little overlap between the emission of [Ir(sppy)_3_]^3−^ and the MLCT absorption band of [Ru(bpy)_3_]^2+^ (Fig. S6†); and (ii) the [Ru(bpy)_3_]^2+^ complex has a lower luminescence quantum yield, but the integrated ECL intensity of the mixture is higher than that of the individual complexes. Moreover, energy transfer was not observed from (Ir(ppy)_3_)* to [Ru(bpy)_2_(L)]^2+^ (where L is a 4,4′-dicarboxamide derivative of 2,2′-bipyridine) in the potential-resolved co-reactant ECL system in acetonitrile.^28^ The enhancement of [Ru(bpy)_3_]^2+^ ECL can therefore be ascribed to electron transfer between the intermediates of the two metal-complexes.

The onset of ECL from both luminophores occurs in the potential region at which both [Ir(sppy)_3_]^3−^ and TPrA are oxidised, and as noted above, the TPrA˙ radical can reduce [Ru(bpy)_3_]^2+^, but not [Ir(sppy)_3_]^3−^. Of the available species under these conditions, the only possible electron transfer between the ground oxidation states of the Ir and Ru complexes is from [Ru(bpy)_3_]^+^ to [Ir(sppy)_3_]^2−^, which is sufficiently energetic to attain [Ru(bpy)_3_]^2+^* (reaction (12); Δ*E*^0^′ ≈ *E*_0–0_), but not [Ir(sppy)_3_]^3−^* (Δ*E*^0^′ < *E*_0–0_).

A similar reaction (1) occurs in the mixed annihilation ECL of [Ru(bpy)_3_]^2+^ and Ir(ppy)_3_ in acetonitrile, which was isolated from other light-producing pathways by alternating between potentials sufficient to oxidise only Ir(ppy)_3_ and reduce only [Ru(bpy)_3_]^2+^.^23,31,33^ The feasibility of generating [Ru(bpy)_3_]^2+^* *via* reaction (12) was verified by selectively reducing [Ru(bpy)_3_]^2+^ and oxidising [Ir(sppy)_3_]^3−^ under annihilation ECL conditions in 80 : 20 acetonitrile : water solution (Fig. S8†). The ECL mechanisms for [Ru(bpy)_3_]^2+^ and [Ir(sppy)_3_]^3−^ with TPrA as co-reactant in aqueous solution described above are summarised in Fig. 2.

12 [Ru(bpy)_3_]^+^ + [Ir(sppy)_3_]^2−^ → [Ru(bpy)_3_]^2+^* + [Ir(sppy)_3_]^3−^

In the mixed co-reactant ECL system, the electrochemically oxidised [Ir(sppy)_3_]^2−^ will not react with the excess TPrA co-reactant, providing a longer lived alternative to TPrA˙^+^ for the generation of [Ru(bpy)_3_]^2+^* *via* reaction (9). This mechanism of enhancement (reaction (12)) would therefore be expected to have a greater effect on the co-reactant ECL of [Ru(bpy)_3_]^2+^ at applied potentials between 0.81 V and 1.06 V (*vs.* SCE), which are sufficient to oxidise TPrA and [Ir(sppy)_3_]^3−^, and generate [Ru(bpy)_3_]^2+^* *via* reaction (9), but not oxidise [Ru(bpy)_3_]^2+^ (which would enable the generation of [Ru(bpy)_3_]^2+^* *via* reactions (6) and (8)). As shown in Fig. 3 and S7,† this is indeed the case. Deconvolution of the contributions from the two luminophores (*e.g.*, Fig. S9c†) shows that by adding 100 μM [Ir(sppy)_3_]^3−^, the co-reactant ECL intensity of [Ru(bpy)_3_]^2+^ increased by 10.8-fold when applying a potential of 0.86 V, but only 1.5-fold at 1.16 V (Fig. S10†). Importantly, the enhanced pathway is the predominant route for ECL detection in magnetic-bead supported assays,^3^ where only a small fraction of the [Ru(bpy)_3_]^2+^ electrochemiluminophore is close enough to the electrode surface to undergo direct oxidation.^5,49^

To summarise the available ECL pathways described above: at potentials between 0.81 V and 1.06 V *vs.* SCE, and at higher potentials if the [Ru(bpy)_3_]^2+^ luminophore is not close enough to the electrode for its direct oxidation, the ECL of [Ru(bpy)_3_]^2+^ can only proceed *via* the remote^5,48^ pathway (Fig. 2a), and regardless of the applied potential, the ECL of [Ir(sppy)_3_]^3−^ can only proceed *via* the direct^5,48^ pathway (Fig. 2b). But when both metal complexes are present under these conditions, the reduced ruthenium complex can also react with the oxidised iridium complex to form [Ru(bpy)_3_]^2+^* (reaction (12)), thereby enhancing the ECL intensity of [Ru(bpy)_3_]^2+^ (Fig. 2c).

**Fig. 4:**
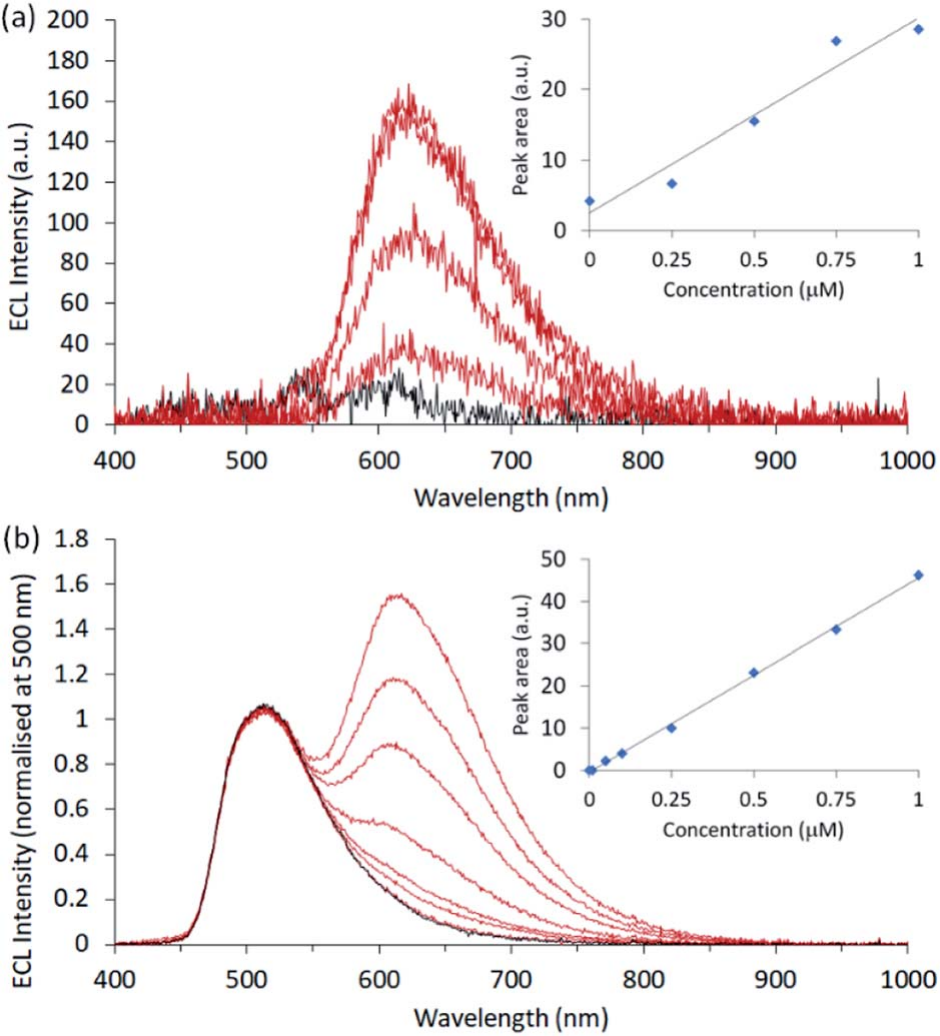
Co-reactant ECL calibrations prepared for [Ru(bpy)_3_]^2+^ in ProCell solution (a) without and (b) with enhancement and internal standardisation by [Ir(sppy)_3_]^3−^ (100 μM). For each experiment, a single chronoamperometric pulse was applied at 0.86 V (*vs.* SCE) for 10 s.

The [Ir(sppy)_3_]^3−^ complex is an excellent candidate for ratiometric ECL detection of [Ru(bpy)_3_]^2+^ under aqueous conditions. It is highly soluble and the spectral distribution of its emission is sufficiently different from the Ru(ii) complex to enable deconvolution of their contributions. Moreover, due to its lower efficiency and strong enhancement of the co-reactant ECL of [Ru(bpy)_3_]^2+^, relatively high concentrations of [Ir(sppy)_3_]^3−^ are required to attain similar intensities. Comparison of the ECL response over the range 1–100 μM [Ir(sppy)_3_]^3−^ in the presence and absence of 0.75 μM [Ru(bpy)_3_]^2+^ (Fig. S10†) shows not only the enhancement of the [Ru(bpy)_3_]^2+^* emission, but also the absence of significant quenching of the Ir(iii) complex ECL, revealing that [Ir(sppy)_3_]^3−^ could serve simultaneously as an enhancer and internal standard. As a preliminary demonstration, we prepared calibrations using seven [Ru(bpy)_3_]^2+^ standards from 10 nM to 1 μM, with and without 100 μM [Ir(sppy)_3_]^3−^. Between each experiment, the ECL cell was disassembled and the working electrode was polished. The slight variation in the re-alignment of electrode and the collimating lens had a significant influence on signal intensity. Under these conditions, the ECL signals without the enhancer exhibited relatively poor precision (RSD of ∼20% at 0.5 μM) and signal-to-noise (Fig. 4a), resulting in a limit of detection of approximately 0.25 μM.

In the presence of the enhancer, the intensity of the [Ru(bpy)_3_]^2+^ emission was markedly increased, but the deleterious variation in signal intensity was compounded by the overlap with the long-wavelength edge of [Ir(sppy)_3_]^3−^. Deconvolution of the emissions could minimise this effect but would not remove the inherent instrumental variability of the [Ru(bpy)_3_]^2+^ signal. However, using [Ir(sppy)_3_]^3−^ as an internal standard (by simply normalising the spectra at 500 nm and subtracting the blank signal; Fig. 4b), the precision (RSD of 2% at 0.25 μM) and linearity (*R*^2^ = 0.9987) were vastly improved, resulting in a limit of detection of 40 nM, without the need for signal deconvolution.

**Fig. 5:**
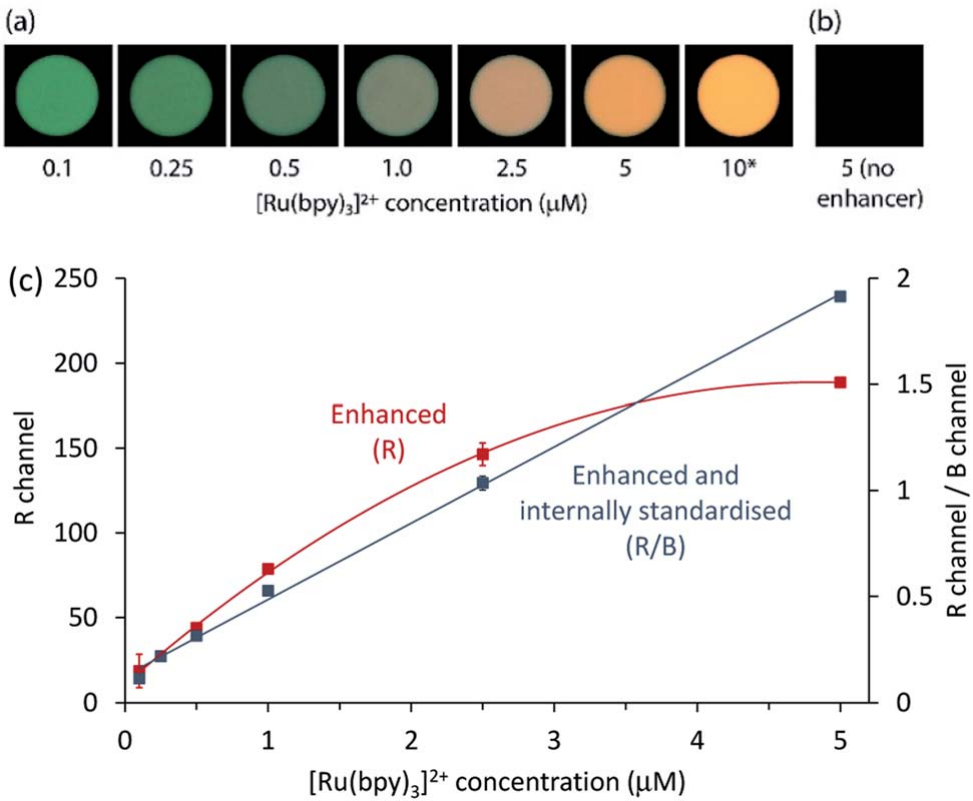
(a) Photographs of ECL at the working electrode surface for different concentrations of [Ru(bpy)_3_]^2+^ in ProCell solution with 100 μM [Ir(sppy)_3_]^3−^, upon application of 0.86 V *vs.* SCE. (b) Photograph of the working electrode for 5 μM [Ru(bpy)_3_]^2+^ in ProCell solution without the enhancer. No ECL could be detected under these conditions. (c) [Ru(bpy)_3_]^2+^ calibrations prepared using the R values (red plot) or the ratio of the R to B values (blue plot) from the RGB data extracted from the images, after initial subtraction of the corresponding value (R) or ratio (R/B) for the blank solution containing the enhancer in ProCell solution, but no [Ru(bpy)_3_]^2+^. Error bars represent ±1 standard deviation (*n* = 3). *The image for 10 μM [Ru(bpy)_3_]^2+^ in ProCell solution with 100 μM [Ir(sppy)_3_]^3−^ was not used in the calibration because the intensity of the emission exceeded the capacity of the R channel. Settings: ISO 10000, *f*/3.5, shutter time 10 s, electrochemical pulse time: 9.5 s with 0.2 s wait between shutter trigger and pulse.

Finally, we sought to apply the enhancement and internal standardisation to co-reactant [Ru(bpy)_3_]^2+^ ECL captured as a digital image. ECL is a promising mode of detection for portable analytical systems integrating consumer devices such as cameras or smartphones,^50^ which can serve as both a power source to initiate the electrochemical reaction and a photodetector to measure the emission.^51–53^ As shown in Fig. 5a, digital photographs of the ECL at the working electrode surface for a solution of 0.1–10 μM [Ru(bpy)_3_]^2+^ in ProCell with 100 μM [Ir(sppy)_3_]^3−^ showed a clear change in colour from that dominated by the green emission of the enhancer to the orange emission of the Ru(ii) complex. The corresponding images of the extracted RGB data are shown in Fig. S11.† Under these conditions, no ECL could be detected from [Ru(bpy)_3_]^2+^ in ProCell solution without the enhancer (*e.g.*, Fig. 5b).

The RGB colour data extracted from the digital images showed a quadratic relationship between the R value and [Ru(bpy)_3_]^2+^ concentration (red plot in Fig. 5c). Using the R/G ratio improved the precision (from 10% to 4% RSD at 0.25 μM), but the relationship was also quadratic (Fig. S12†), due to the significant contribution of both luminophores to the R and G channels. As the [Ru(bpy)_3_]^2+^ emission had a relatively minor influence on the B value, the R/B ratio provided effective internal standardisation from [Ir(sppy)_3_]^3−^, generating a linear calibration (*R*^2^ = 0.9982), as depicted by the blue plot in Fig. 5c. The greater precision of the R/B calibration (3% RSD at 0.25 μM) resulted in a lower limit of detection (80 nM; 3σ) than that prepared using R values only (0.4 μM).

## Experimental section

### Chemicals and general details

Reagents and solvents were purchased from various commercial sources and used without further purification. Tris(2,2′-bipyridine)ruthenium(ii) dichloride hexahydrate ([Ru(bpy)]_3_Cl_2_·6H_2_O) was purchased from Strem (USA). [Ir(df-ppy)_2_(pt-TEG)]Cl was prepared as previously described.^20,52^ Potassium phosphate monobasic and dibasic salt, sodium chloride, TPrA and *fac*-Ir(ppy)_3_ were purchased from Sigma-Aldrich Australia. ProCell buffer solution containing the TPrA co-reactant was purchased from Roche Australia. NMR spectra were acquired on a Bruker Biospin AV400 spectrometer. ^1^H NMR spectra were acquired at 400 MHz, ^13^C{^1^H} NMR spectra were acquired at 101 MHz. All NMR spectra were recorded at 298 K. Chemical shifts were referenced to residual solvent peaks and are quoted in terms of parts per million (ppm), relative to tetramethylsilane (Si(CH_3_)_4_).

### Synthesis

The [Ir(sppy)_3_]^3−^ complex was synthesised according to the previously reported procedure^37^ with some modification. A mixture of concentrated sulfuric acid (99 mg, 1.0 mmol) and trifluoroacetic anhydride (10 mL) was set stirring at ambient temperature in an N_2_ atmosphere. After 1 h, a solution of *fac*-Ir(ppy)_3_ (195 mg, 0.3 mmol) in dichloromethane (40 mL) was added dropwise and the reaction mixture was stirred for 24 h. The solvent was removed by evaporation under a stream of N_2(g)_ followed by addition of a few drops of saturated aqueous sodium carbonate to the residue. The mixture was lyophilised and the solid was washed several times with methanol. The volume of methanol was reduced under a stream of N_2(g)_ and acetonitrile was added then the mixture was stored at −20 °C and a precipitate was observed. The precipitate was isolated by filtration and washed with acetonitrile then dried at the pump, and purified by column chromatography (SiO_2_, 30% NH_4_OH/methanol/acetonitrile, 1/5/50) to afford a yellow powder (75 mg, 0.08 mmol, 27%). NMR spectra matched those previously reported for this compound.^37^

### Luminescence measurements

Photoluminescence (PL) spectra were obtained using a Cary Eclipse fluorescence spectrophotometer and UV-visible absorbance spectra were obtained using a Cary 300 spectrophotometer from 10 μM metal complex in deionised water using a quartz cuvette with a cell path length of 1 cm. For the PL spectra, the PMT voltage was 600 V and the excitation wavelength was 271 nm for [Ir(sppy)_3_]^3−^, 620 V and 258 nm for [Ir(ppy)_2_(pt-TEG)]^+^, and 700 V and 285 nm for [Ru(bpy)_3_]^2+^. The data interval for all collected spectra was 1 nm with an excitation bandpass filter of 250–395 nm for all analytes. The emission filter was 430–1100 nm for [Ru(bpy)_3_]^2+^ and [Ir(sppy)_3_]^3−^, and 360–1100 nm for [Ir(ppy)_2_(pt-TEG)]^+^. Low temperature PL spectra were obtained using 5 μM metal complex in an ethanol : methanol (4 : 1) mixture that was cooled to 85 K using an OptistatDN Variable Temperature Liquid Nitrogen Cryostat equipped with custom-made quartz sample holder.^54^ The temperature of 85 K was used to avoid damage to the spectroscopic cuvettes which occurred at 77 K.^55^ We previously found no significant difference in *λ*_max_ in the spectra of [Ru(bpy)_3_]^2+^ and Ir(ppy)_3_ measured at these two temperatures under these instrumental conditions.^54^ All PL emission spectra were corrected for the change in instrumental sensitivity over the wavelength range by multiplication with correction curves established using a quartz halogen tungsten lamp.

### Electrochemistry and ECL

We used a previously described custom cell design^31^ with glassy carbon working, platinum wire counter (CH Instruments) and leakless Ag/AgCl reference (model ET069; eDAQ Australia) electrodes to collect all electrochemical and ECL data. We polished the glassy carbon electrode using 1 μm and 0.05 μm alumina polishing powder (CH Instruments) followed by sonicating in ethanol prior to each experiment. An Autolab PGSTAT204 or Autolab PGSTAT128N was used for all electrochemical measurements. Potentials were referenced to SCE from Ag/AgCl (3.5 M KCl) by subtracting 40 mV.^45^ The cell was interfaced with a photomultiplier tube (PMT, extended-range trialkali S20 PMT, ET Enterprises model 9828B) to measure ECL intensity, a charge coupled device (CCD, QEPro, Ocean Optics) to obtain ECL spectra, or a digital camera (Canon EOS 6D DSLR camera, fitted with a Tonika AT-X PRO MACRO 100 mm f/2.8 D lens) to collect images of the ECL at the working electrode surface, as previously described.^29,31,53^ ECL experiments were conducted in ProCell or 0.1 M phosphate buffer solution (PBS) with 100 mM TPrA with pH adjusted using NaOH or HCl to obtain the desired pH. When required, the ECL spectra were deconvoluted^31^ into the two characteristic emission bands (defined by the ECL spectrum of each metal complex) using the Solver function of Excel, where the concentrations of [Ru(bpy)_3_]^2+^ (*c*_Ru_) and [Ir(sppy)_3_]^3−^ (*c*_Ir_) were solved by minimising the sum of the squared differences between the model (*I*_total_ = *c*_Ru_*I*_Ru_ + *c*_Ir_*I*_Ir_) and the measured intensity (*I*_meas_) at each wavelength. RBG data were extracted from the digital images of ECL at the working electrode surface using ImageJ (https://imagej.nih.gov/ij/).^56^Fig. 3 and S5† were prepared using the ‘Contour’ plot type in OriginPro, Version 2021b (OriginLab Corporation, Northampton, MA, USA).

## Conclusions

The water-soluble [Ir(sppy)_3_]^3−^ electrochemiluminophore not only enhanced the co-reactant ECL of [Ru(bpy)_3_]^2+^ at 0.86 V (*vs.* SCE) by over an order of magnitude, but also served as an effective internal standard, providing superior precision. The ability to apply these advances in sensitivity and precision to ECL measured by digital photography through the ratio of R to B colour data is particularly promising for the development of low-cost, portable analytical devices. Furthermore, considering the importance of Ir(ppy)_3_ in the fundamental development of multi-colour ECL in organic solvents, these findings are an important step in its translation to analytical applications in aqueous solution. More generally, this synthetic approach is a convenient alternative to the synthesis of Ir(iii) complexes from novel ligands containing polar functional groups that could enable a wide range of promising electrochemiluminophores to be utilised under aqueous conditions.

## Data availability

The data supporting the findings of this study are included in the main text and in the ESI file.†

## Author contributions

Emily Kerr: Conceptualization, Methodology, Investigation, Validation, Formal analysis, Writing – original draft, Writing – review & editing, Visualization, Supervision, Project administration, Funding acquisition. David Hayne: Methodology, Investigation, Supervision, Writing – original draft, Writing – review & editing. Lachlan Soulsby: Methodology, Investigation, Writing – review & editing. Joseph Bawden: Investigation, Writing – original draft, Writing – review & editing. Steven Blom: Investigation, Writing – review & editing. Egan Doeven: Methodology, Supervision, Writing – review & editing. Luke Henderson: Methodology, Supervision, Funding acquisition, Writing – review & editing. Conor Hogan: Methodology, Writing – review & editing. Paul Francis: Conceptualization, Methodology, Validation, Resources, Formal analysis, Writing – original draft, Writing – review & editing, Visualization, Supervision, Project administration, Funding acquisition.

## Conflicts of interest

There are no conflicts to declare.

## Supplementary Material

SC-013-D1SC05609C-s001
