# Supplementary material for: A redox-mediator pathway for enhanced multi-colour electrochemiluminescence in aqueous solution
Source: Chem Sci. 2021 Dec 15;13(2):469–77. doi: 10.1039/d1sc05609c (PMC8729815; doi:10.1039/d1sc05609c)
Supplement: SC-013-D1SC05609C-s001 [file SC-013-D1SC05609C-s001.pdf]

Electronic Supplementary Material (ESI) for Chemical Science.
This journal is © The Royal Society of Chemistry 2021



## Supplementary Information

**Fig. S1**. Cyclic Voltammetry (scan rate: 0.1 V s$^{-1}$). (a) [Ir(sppy)$_3$]$^{3-}$, (b) [Ru(bpy)$_3$]$^{2+}$ and (c) [Ir(ppy)$_2$(pt-TEG)]$^{+}$, at 1 mM in 0.1 M PBS (pH 7.5). (d) 100 mM TPrA in 0.1 M PBS (pH 7.5). (e) ProCell solution. The $E^{0\prime}$ (M$^+$/M) (*vs* SCE) for [Ir(sppy)$_3$]$^{3-}$, [Ru(bpy)$_3$]$^{2+}$ and [Ir(ppy)$_2$(pt-TEG)]$^{+}$ (Table 1) were in good agreement with previously reported values.[1]

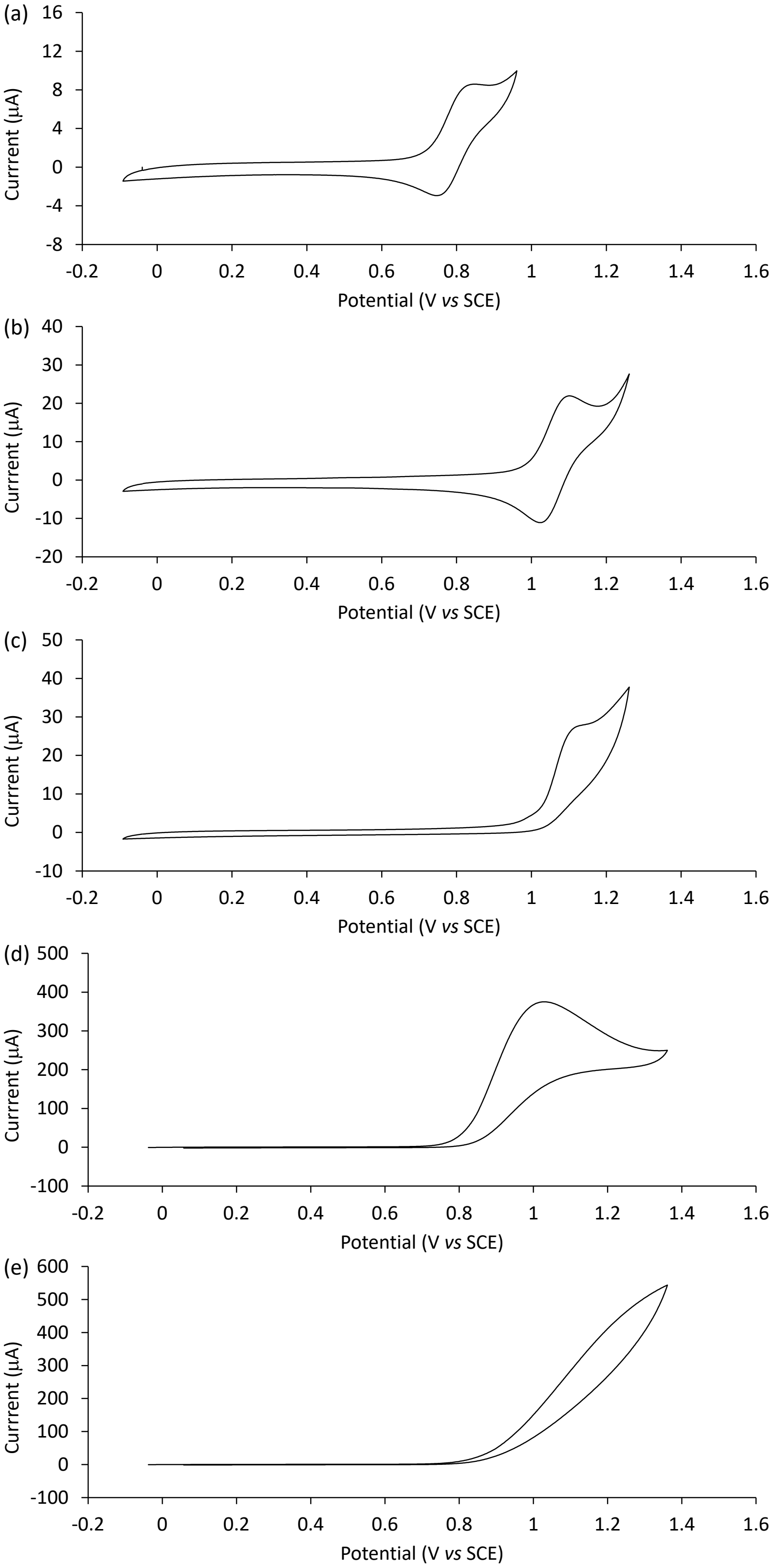

**Fig. S2**. (a) UV-visible absorption spectra, and (b) photoluminescence emission spectra at ambient temperature, of $[Ru(bpy)_3]^{2+}$ (red plot), $[Ir(sppy)_3]^{3-}$ (green plot), and $[Ir(ppy)_2(pt\text{-}TEG)]^+$ (blue plot), at 10 μM in deionised (Milli-Q) water, and $Ir(ppy)_3$ (black plot) at 10 μM in acetonitrile. (c) photoluminescence emission spectra of the same metal complexes at 5 μM in 4:1 ethanol:methanol at low temperature (85 K). All emission spectra were corrected for the variation in instrument sensitivity over the wavelength range and then normalised.

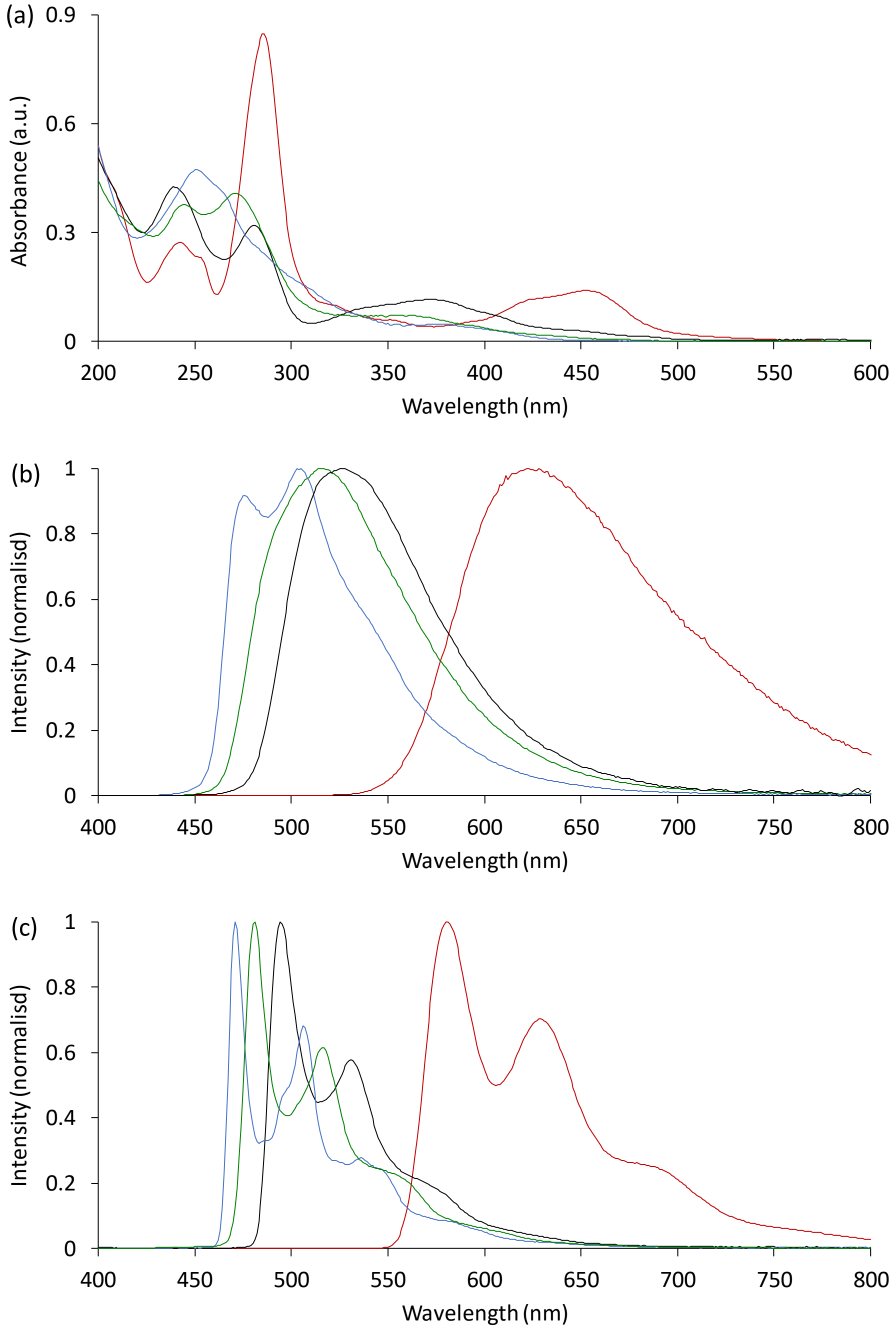

**Fig. S3**. (a) Co-reactant ECL spectra of 10 µM $[Ru(bpy)_3]^{2+}$ (red plot), 100 µM $[Ir(sppy)_3]^{3-}$ (green plot) and 10 µM $[Ir(ppy)_2(pt\text{-}TEG)]^{+}$ (blue plot) in ProCell solution. (b) Photographs of the co-reactant ECL at the surface of the working electrode at the stated applied potential *vs* SCE (ISO 3200, f/ 2.8, Exposure times of 16 s, 0.2 s and 0.2 s for the $[Ir(sppy)_3]^{3-}$, $[Ir(ppy)_2(pt\text{-}TEG)]^{+}$ and $[Ru(bpy)_3]^{2+}$; 0.2 mM in 0.1 M PBS (pH 7.5) with 100 mM TPrA). (c) CIE plot of the ECL emission colour of the three luminophores (CIE 1931 2° observer).

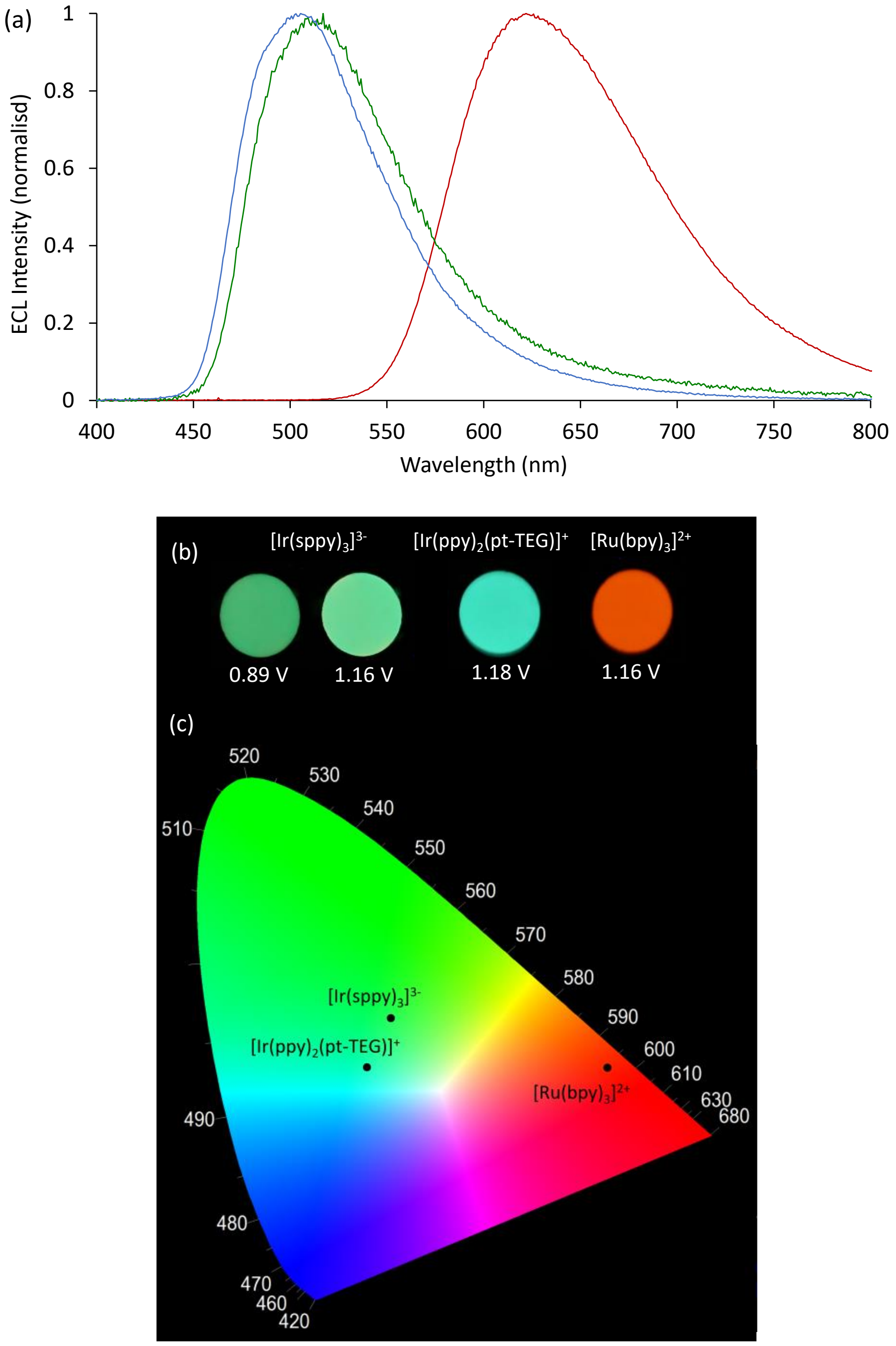

**Fig. S4**. ECL spectra at 0.76 V (grey plot) and 0.81 V (blue plot) *vs* SCE (10 s pulse) for a mixture of $[Ir(sppy)_3]^{3-}$ (150 µM) and $[Ru(bpy)_3]^{2+}$ (0.75 µM) in ProCell, showing the approximate ECL on-set potential for both luminophores.

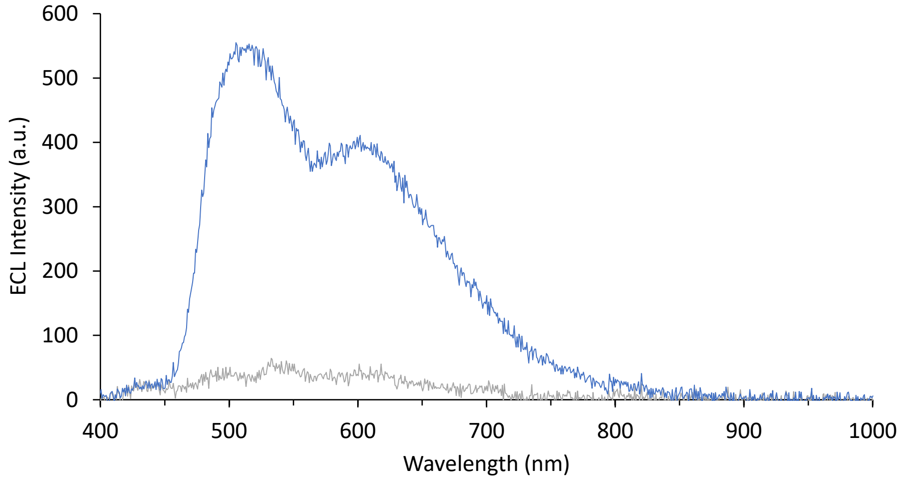


**Fig. S5.** Contour plot of ECL *vs* wavelength and applied potential for $[Ir(sppy)_3]^{3-}$ (150 µM) in ProCell solution, prepared by applying the potentials (10 s chronoamperometric pulses) in 50 mV intervals. Fig. 3 shows the contour plots for a mixture of $[Ru(bpy)_3]^{2+}$ (0.75 µM) and $[Ir(sppy)_3]^{3-}$ (150 µM), or $[Ru(bpy)_3]^{2+}$ (0.75 µM) alone.

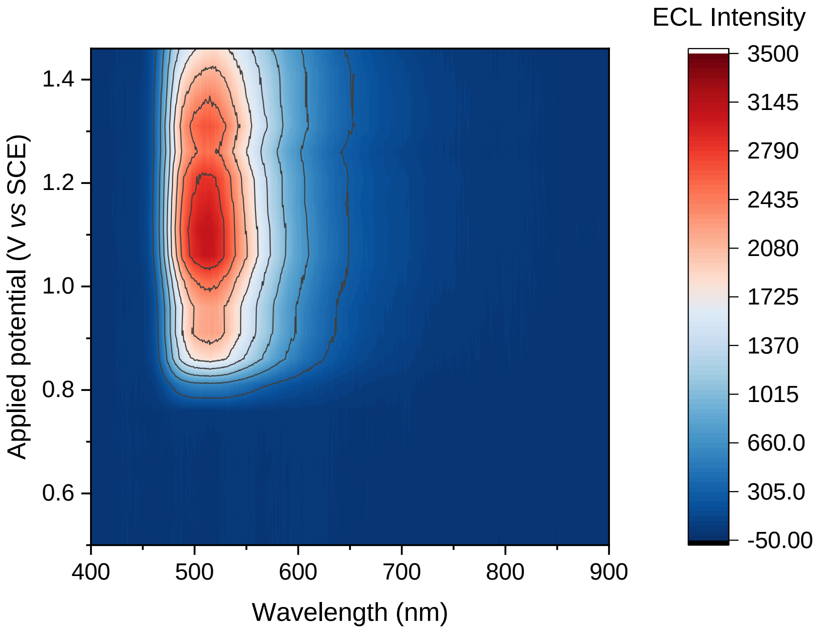

**Fig. S6**. UV-visible absorption spectra of $[Ru(bpy)_3]^{2+}$ (red plot) and photoluminescence emission spectra of $[Ir(sppy)_3]^{3-}$ (green plot), both at a metal complex concentration of 10 µM in deionised (Milli-Q) water at ambient temperature.

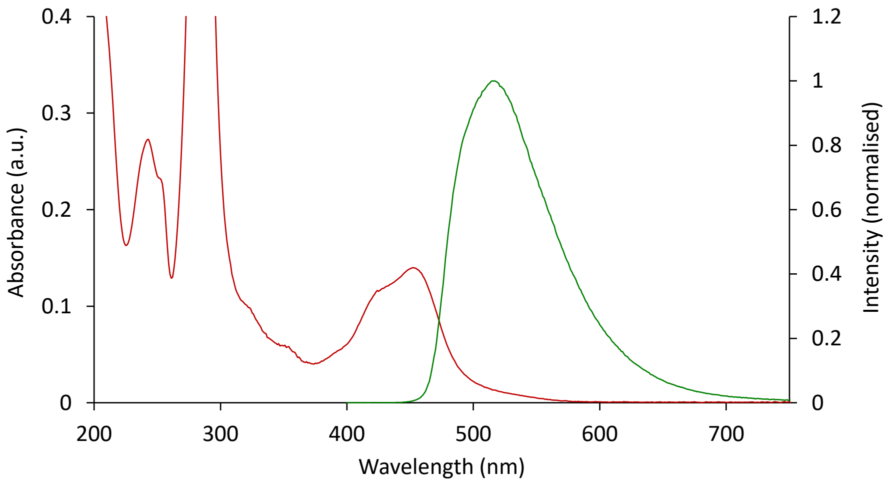


**Fig. S7.** ECL intensity at 620 nm *vs* applied potential (10 s chronoamperometric pulses at 50 mV intervals) for 0.75 µM $[Ru(bpy)_3]^{2+}$ (red plot) or a mixture of 0.75 µM $[Ru(bpy)_3]^{2+}$ and 150 µM $[Ir(sppy)_3]^{3-}$ (black plot), in ProCell solution, extracted from the data shown in Fig. 3. The grey plot shows the ECL intensity at 620 nm *vs* applied potential for the mixture of 0.75 µM $[Ru(bpy)_3]^{2+}$ and 150 µM $[Ir(sppy)_3]^{3-}$, after subtracting the intensity for 150 µM $[Ir(sppy)_3]^{3-}$ (extracted from the data depicted in Fig. S5). The vertical dashed lines show the $E^{0\prime}$ for $[Ir(sppy)_3]^{3-}$, TPrA and $[Ru(bpy)_3]^{2+}$ in aqueous solution.

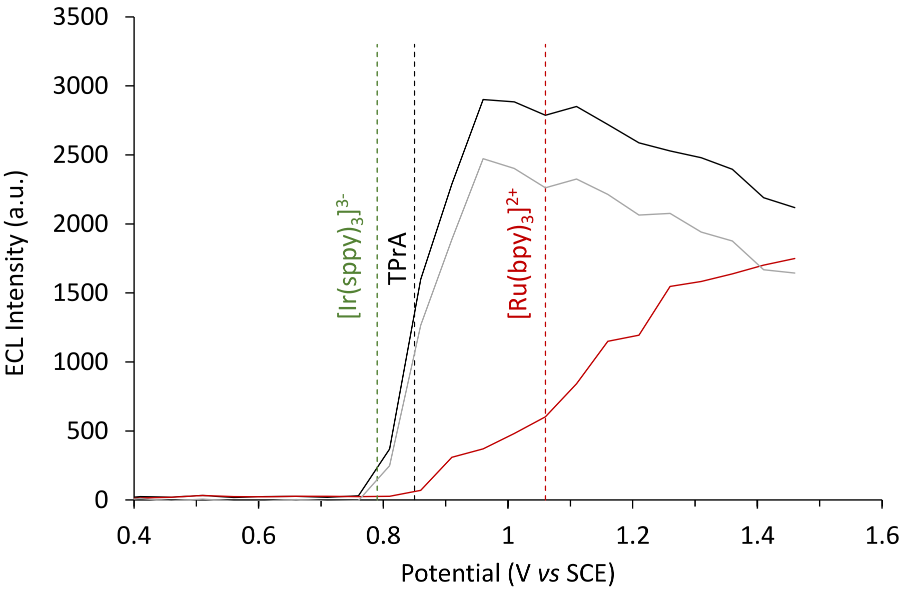

**Fig. S8**. Red plot: 'Mixed annihilation ECL' of 10 µM $[Ru(bpy)_3]^{2+}$ and 250 µM $[Ir(sppy)_3]^{3-}$ in 80:20 acetonitrile:water solution containing 0.1 M $TBAPF_6$ electrolyte, involving alternating application of −1.44 V *vs* SCE, which only reduces the Ru(II) complex, and 0.92 V *vs* SCE, which oxidises only the Ir(III) complex. The spectral distribution matches the characteristic emission of $[Ru(bpy)_3]^{2+}$*. Grey plot: no ECL was observed when the same potentials were applied in the absence of $[Ir(sppy)_3]^{3-}$. In each experiment, 50 x 0.1 s pulses were applied.

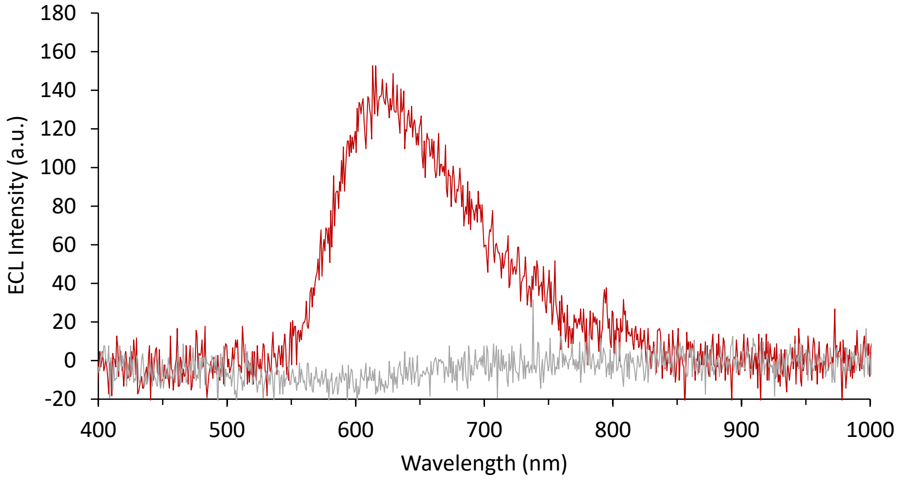

**Fig. S9**. ECL spectra for 0.75 μM $[Ru(bpy)_3]^{2+}$ and 100 μM $[Ir(sppy)_3]^{3-}$ (blue plots) in ProCell solution, at an applied potential of (a) 1.16 V or (b) 0.86 V *vs* SCE, compared to the individual electrochemiluminophores under the same conditions (red and green plots, respectively). (c) Deconvolution of the mixture (*i.e.*, the blue plot of Fig. S9b), where the red and green lines show the deconvoluted emissions from the $[Ru(bpy)_3]^{2+}$ and $[Ir(sppy)_3]^{3-}$ luminophores, and the black line is the sum of the two components showing the excellent fit with the raw data. For clarity, spectra shown in this figure were smoothed by adjacent averaging (15 pt).

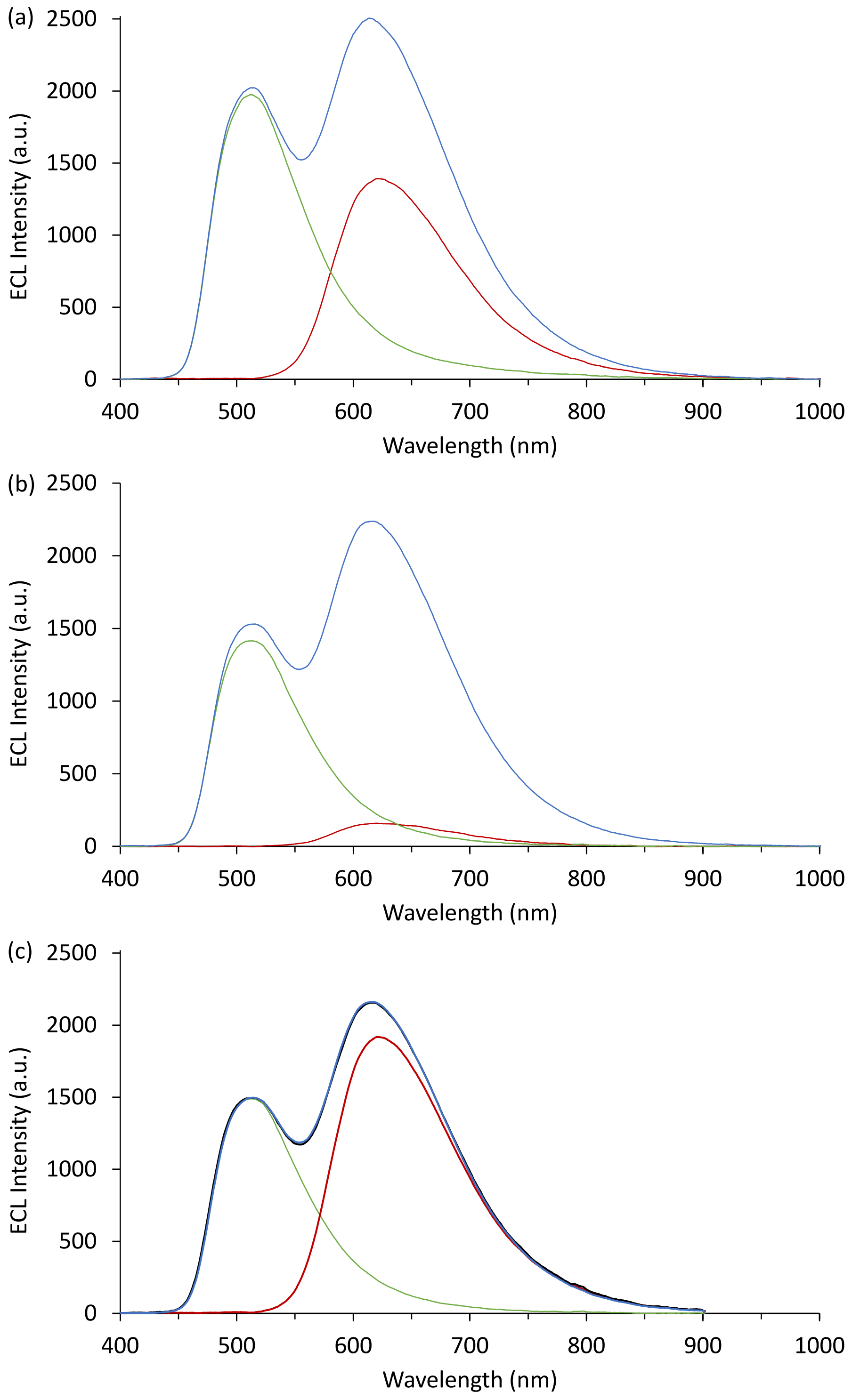

**Fig. S10**. ECL spectra generated with an applied potential of (a) 1.16 V and (b) 0.86 V *vs* SCE for varying concentrations of $[Ir(sppy)_3]^{3-}$ (1, 5, 25, 50 or 100 µM) in ProCell solution, without (green plots) and with (blue plots) 0.75 µM $[Ru(bpy)_3]^{2+}$. The black plots show the ECL spectra for 0.75 µM $[Ru(bpy)_3]^{2+}$ in ProCell solution without $[Ir(sppy)_3]^{3-}$. Spectra were smoothed by adjacent averaging (15 pt). (c) Relative ECL intensity of $[Ru(bpy)_3]^{2+}$ in the experiments depicted in Fig. S10a and S10b, established by deconvolution of the emission spectra from both luminophores (for example, see Fig. S9c), relative to the emission at each applied potential in the absence of $[Ir(sppy)_3]^{3-}$.

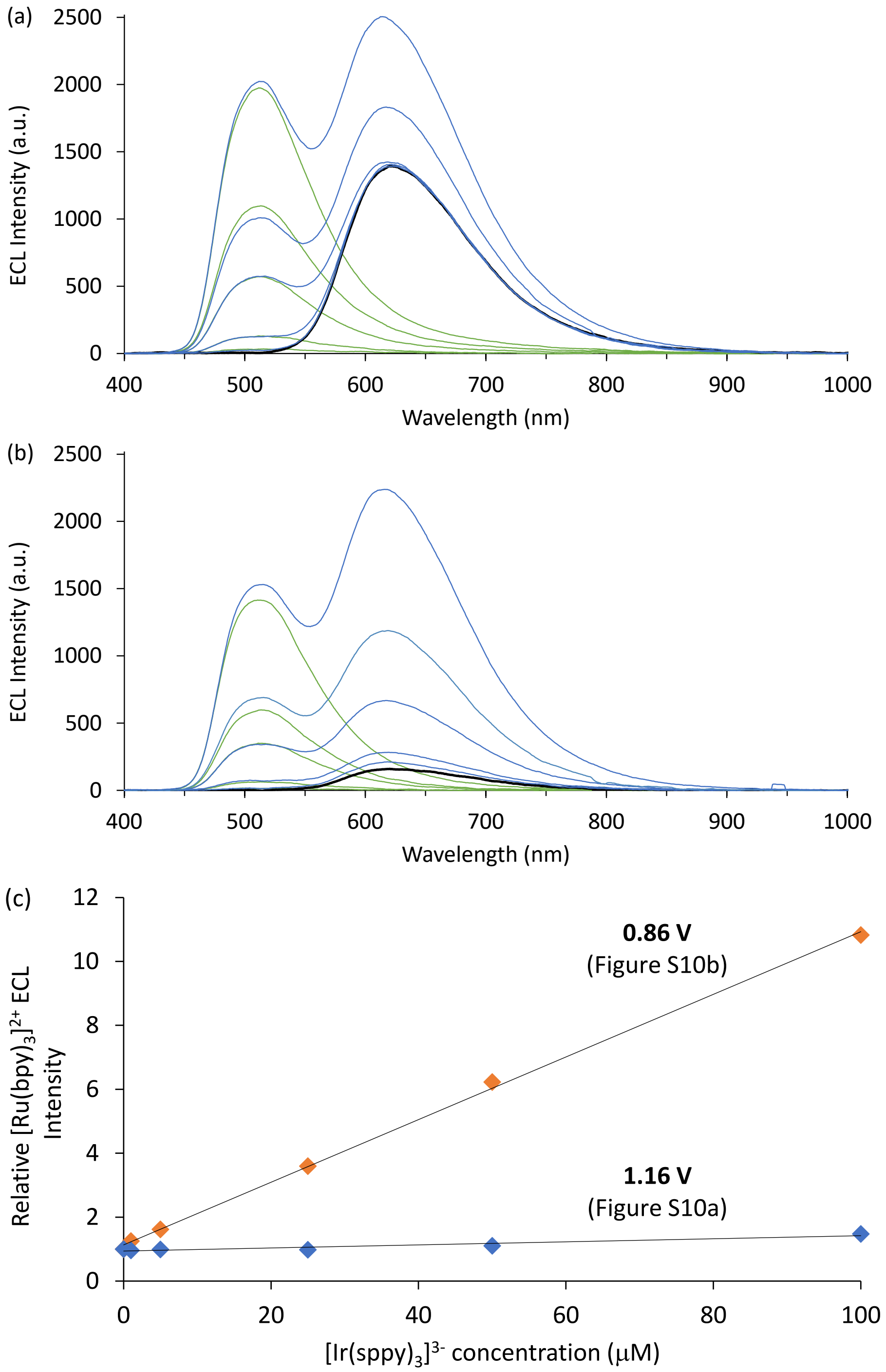

**Fig. S11**. Photographs and corresponding images of the extracted RGB data of the ECL at the working electrode surface for 0.1-10 μM $[Ru(bpy)_3]^{2+}$ in ProCell solution with 100 μM $[Ir(sppy)_3]^{3-}$ upon application of 0.86 V *vs* SCE. The rightmost column shows the data for 5 μM $[Ru(bpy)_3]^{2+}$ in ProCell solution without the $[Ir(sppy)_3]^{3-}$ enhancer, under the same experimental conditions. Settings: ISO 10,000, f/ 3.5, shutter time 10 s, electrochemical pulse time: 9.5 s with 0.2 s wait between shutter trigger and chronoamperometric pulse.

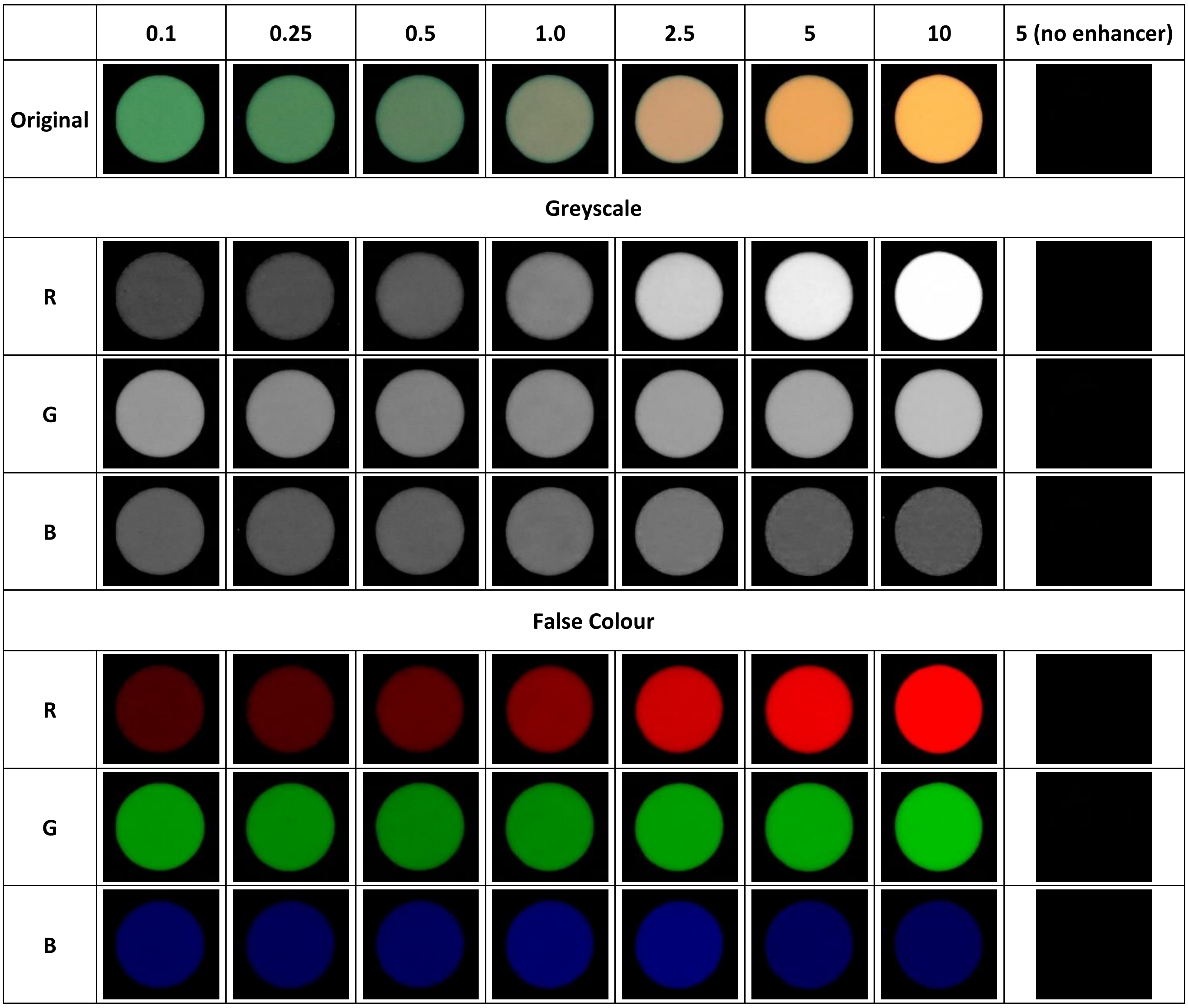

**Fig. S12**. $[Ru(bpy)_3]^{2+}$ calibrations prepared using the R values (red plot) or the ratio of the R to G values from the RGB data extracted from the images (Fig. S11), after initial subtraction of the corresponding value (R) or ratio (R/G) for the blank solution containing the enhancer in ProCell, but no $[Ru(bpy)_3]^{2+}$. Error bars represent ±1 standard deviation (n = 3).

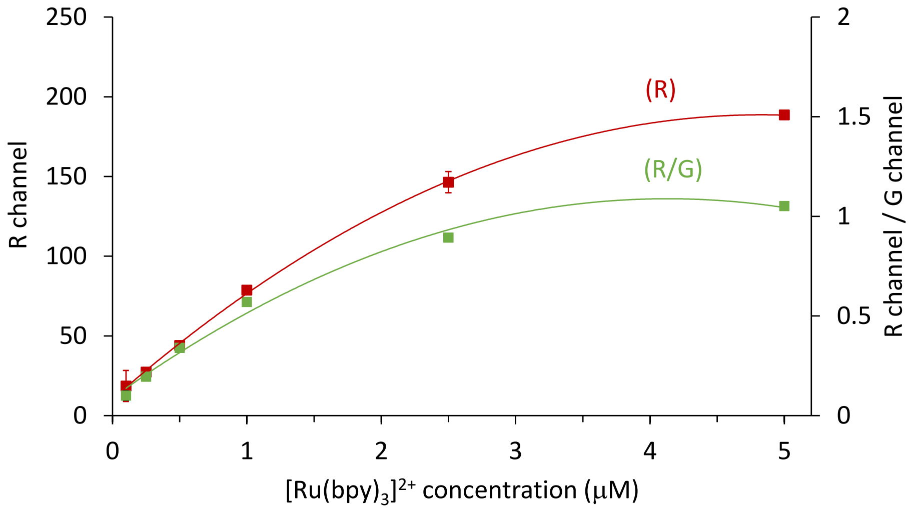


## References


1. X. Guo, Y. Okamoto, M. R. Schreier, T. R. Ward and O. S. Wenger, *Chem. Sci.*, 2018, **9**, 5052-5056; A. Fiorani, G. Valenti, Irkham, F. Paolucci and Y. Einaga, *Phys. Chem. Chem. Phys.*, 2020, **22**, 15413-15417; L. Chen, D. J. Hayne, E. H. Doeven, J. Agugiaro, D. J. D. Wilson, L. C. Henderson, T. U. Connell, Y. H. Nai, R. Alexander, S. Carrara, C. F. Hogan, P. S. Donnelly and P. S. Francis, *Chem. Sci.*, 2019, **10**, 8654-8667.
